# Acrodermatitis Enteropathica in the Peruvian Amazon: A Case of Zinc-Deficiency in a Resource-Limited Setting

**DOI:** 10.51894/001c.144414

**Published:** 2025-09-30

**Authors:** Yuri Alonte

**Affiliations:** 1 College of Osteopathic Medicine Michigan State University

## 61

### INTRODUCTION

Acrodermatitis Enteropathica (AE) is a rare genetic autosomal recessive disorder with a global incidence rate of 1:500,000 live births, affecting populations independent of ethnicity or sex. AE is caused by zinc deficiency, with a classic triad of peri-acral and periorificial dermatitis, diarrhea, and alopecia. The genetic form of AE results from a mutation in the SLC39A4 gene, which encodes a zinc transporter required for intestinal absorption. An acquired form of AE can result due to reduced intake, increased demand, or malabsorption. During development, zinc is essential for synaptic transmission and neuronal plasticity, with deficiencies linked to cognitive impairment and neurodegenerative diseases.

### CASE DESCRIPTION

A 20-month-old patient presented to our clinic in Iquitos, Peru with a one-week history of diarrhea and three-day history of a rash in the diaper area. The diarrhea was watery and bloody, accompanied by fever, decreased appetite, and noticeable weight loss. Prior to presentation, the patient completed a seven-day course of Trimethoprim/Sulfamethoxazole, likely for suspected bacterial cause. Despite resolution of diarrhea and fever, poor appetite and weight loss persisted. The patient had not been consuming formula and discontinued breastfeeding one month prior. Physical examination revealed sharply demarcated, scaly, erythematous plaques without significant moisture, extending from the inguinal region beyond the diaper area. Based on the clinical presentation and exam findings, AE was suspected. The differential diagnosis included impetigo, atopic dermatitis, candidiasis, and erythrasma.

### DISCUSSION/CONCLUSIONS

The patient was started on children’s multivitamins and zinc supplementation. While AE diagnosis ideally involves a plasma zinc measurement, clinical assessment is instrumental in resource-limited settings. The absence of yellow crusting excluded impetigo, the lack of involvement of the flexor surfaces of the extremities reduced suspicion of atopic dermatitis, the absence of moisture lowered the suspicion for candidiasis, and a lack of reddish-brown patch coloration ruled out erythrasma. Given the role of zinc in neuronal development, recognizing the cutaneous manifestations of AE is critical, particularly in resource-limited settings where diagnostic delays may occur due to limited access to genetic and laboratory testing. Witnessed verbal patient consent was given from the patient’s mother in native language for case report.

